# Revisiting Migraine: The Evolving Pathophysiology and the Expanding Management Armamentarium

**DOI:** 10.7759/cureus.34553

**Published:** 2023-02-02

**Authors:** Prathamesh Gawde, Harsh Shah, Harsh Patel, Koppineedi S Bharathi, Neil Patel, Yashendra Sethi, Nirja Kaka

**Affiliations:** 1 Medicine and Surgery, Lokmanya Tilak Municipal Medical College, Mumbai, IND; 2 Medicine and Surgery, Pandit Deendayal Upadhyay Medical College, Rajkot, IND; 3 Internal Medicine, GMERS (Gujarat Medical Education and Research Society) Medical College, Sola, Ahmedabad, IND; 4 Medicine and Surgery, Government Medical College, Surat, Surat, IND; 5 Medicine and Surgery, GMERS (Gujarat Medical Education and Research Society) Medical College, Himmatnagar, IND; 6 Medicine and Surgery, Government Doon Medical College, Dehradun, IND

**Keywords:** pain management, aura, photophobia, neuromodulation, headache, migraine

## Abstract

Migraine affects about one billion people worldwide yearly and is one of the most common neurologic illnesses, with a high prevalence and morbidity, particularly among young adults and females. Migraine is associated with many comorbidities, including stress, sleep difficulties, and suicidal ideation. Migraine, despite its widespread occurrence, is underdiagnosed and undertreated. Because of the complicated and primarily unknown mechanisms of migraine formation, several social and biological risk factors, such as hormone imbalances, genetic and epigenetic impacts, and cardiovascular, neurological, and autoimmune illnesses, have been proposed. Through the mid-20th century diversion of the now-defunct vascular theory, the pathophysiology of migraine has developed from a historical study of the "humours" to a distinct entity as a neurological disorder. The range of therapeutic targets has broadened significantly, increasing the number of specialized clinical trials. Understanding the biology of migraine through careful research has resulted in the identification of major therapeutic classes: (i) triptans, serotonin 5-HT1B/1D receptor agonists, (ii) gepants, calcitonin gene-related peptide (CGRP) receptor antagonists, (iii) ditans, 5-HT1F receptor agonists, (iv) CGRP monoclonal antibodies, and (v) glurants, mGlu5 modulators, with further targets being explored. This review provides a comprehensive overview of the most recent literature on epidemiology and risk factors and exposes knowledge gaps.

## Introduction and background

Recurrent, episodic, and incapacitating headaches, called migraines, sometimes come with various sensorial and motor abnormalities. A neurovascular cause is primarily attributed to the pathogenesis of migraine [1]. Children, adults, and the elderly are all equally prone to this persistent headache condition. When it comes to the clinical appearance of migraine, there is an age-dependent progression from a few times a year in infancy to a few times per week in adulthood, especially in females. Migraine can be episodic or persistent, with or without an aura, and with or without warning symptoms [2]. Once the headache starts, it usually throbs, gets worse with an increase in intracranial pressure, and manifests with other migraine-related symptoms, including premonitory symptoms, nausea, vomiting, photophobia, phonophobia, osmophobia, allodynia, and cranial autonomic symptoms (at least one). According to the International Classification of Headache Disorders (ICHD), these symptoms are all indicators of core migraine symptoms. The characteristics of migraine attacks vary greatly not only between patients but also within the same patient with subsequent episodes [3].

Migraine has been listed as the third most common disorder globally by the Global Burden of Disease Study (GBD) 2010 and as the third-highest cause of disability in the world for both sexes under 50 years old in GBD 2015 [4]. Migraine is the second most incapacitating condition worldwide, after low back pain, according to the GBD 2016 data [5]. The highest migraine prevalence is in the age group of 18-44 years. As people get older, migraines become less common. The prevalence is 15.9% for people aged 45-64 years old, 7.3% for people aged 65-74 years, and 5.1% for people aged 75 and older. Burch et al. found disease variations based on gender, ethnicity, employment position, earnings, and insurance type. Men were less likely than women to suffer from migraines. According to the National Health Interview Study, the prevalence of migraines or other severe headaches is 15.3% overall, 20.7% among women, and 9.7% among men [6].

The potential causes of migraine have been studied, and it is generally agreed that hereditary and environmental variables affect how the trigeminovascular system is activated (for example, by circulating pro-inflammatory chemicals and an oxidative state) [7]. The three main forms of clinically relevant migraines are migraine without aura, migraine with aura, and chronic migraine [8]. Bouts of migraine with aura occur repeatedly and continue for a few minutes. The auras are neurological, gastrointestinal, and autonomic sensory symptoms that are completely reversible and can happen before or during a migraine attack. Flashes of light, blind spots, tingling in the hands or face, impaired speech, and gradually worsening motor problems are some of these symptoms [9]. A migraine without aura is a recurrent headache attack lasting 4-72 hours; it typically is unilateral, pulsating, moderate to severe in severity, worsened by physical activity, and is accompanied by nausea, photophobia, and phonophobia. Chronic migraine is defined as a headache that lasts longer than three months, occurs 15 or more days per month, and exhibits migraine-like symptoms at least eight days per month [10].

Some of the comorbidities linked to migraine include cardiovascular diseases (stroke and myocardial infarction), mental health issues (depression, anxiety, panic disorder, bipolar disorder, personality disorders, and suicide attempts), sleep issues (insomnia, restless leg syndrome, sleep apnea, poor sleep quality, and duration), inflammatory ailments (such as allergic rhinitis and asthma), and chronic pain problems (such as osteoarthritis). Comorbid conditions are known to increase the likelihood of developing chronic migraine. Recent research has demonstrated a link between the accumulation of comorbidities or "multimorbidity," pharmaceutical abuse, and newly onset chronic migraine [11]. According to data from a primary care database research in Scotland, patients with chronic migraine are more likely to have comorbidities than control patients [12].

This review aims to broaden the understanding of migraine pathophysiology and management while filling the gaps in the present pool of information. The authors discuss various approaches to migraine diagnosis. Additionally, they review the newer drugs that have been discovered and some older drugs that have been repurposed for migraine management. This review will also shed light upon the emerging and prevailing therapies for treating migraine.

## Review

Classification of migraine

According to the ICHD (Table 1), the classification is hierarchical, ranging from the first to the fifth digit. One's first impression is based on the group to which the patient belongs. Then, information is acquired that enables a more thorough diagnosis. Only first- or second-digit diagnoses are typically used in general practice, whereas fourth- or fifth-digit diagnoses are appropriate in specialist practice and headache centers.

**Table 1:** ICHD-3 Code ICHD: International Classification of Headache Disorders

ICHD-3 Code	Diagnosis
1.	Migraine
1.1	Migraine without aura
1.2	Migraine with aura
1.3	Chronic migraine
1.4	Complications of migraine
1.5	Probable migraine
1.6	Episodic syndromes that may be associated with migraine

History and evolution of treatment modalities

**Figure 1:**
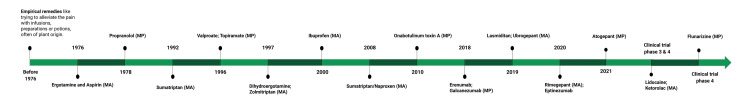
Timeline showing developments in management options for migraine MA: migraine abortive; MP: migraine prophylaxis

Migraine treatment has evolved over the years (Figure 1). Empirical treatments tried to treat the pain with infusions, mixtures, or potions, frequently made from plants. Ergotamine and aspirin were discovered later in the 19th century, changing treatment strategies. Ergotamine and other ergot derivatives were formulated in the early 20th century and were advised for treating migraines, but only for the initial attack. Amitriptyline, beta-blockers, and pizotifen are a few examples of novel compounds that were created between 1950 and 1960 to prevent recurrent episodes. However, the trial findings were frequently inconclusive, and many side effects hindered the treatment. Moskovitz postulated the participation of the trigeminovascular system later in the 1980s. Triptans, the first migraine-specific medicines, were created shortly after that [13].

Acute Migraine Treatment

Acute migraine medications generally fall under general analgesics and drugs specifically designed to treat acute migraine. 5-HT (serotonin) receptor agonists and medications that block calcitonin gene-related peptide (CGRP) are among the drugs specifically designed to treat acute migraine. Cyclooxygenase (COX) inhibitors are among the common analgesics that are used to treat a variety of pain-related illnesses. Acute therapy techniques might be either stratified or step-based. The severity of the attack and patient-specific circumstances determine the method of treatment. Patients in step care start with a nonspecific therapy (i.e., a simple or combination analgesic). Patients revisit their clinician for therapy escalation after treating numerous attacks if the treatment outcome is unsatisfactory. Until a positive treatment outcome is obtained, the process is repeated. In stratified care, the initial course of therapy is decided upon after assessing the patient's treatment requirements and the Migraine Disability Assessment (MIDAS) score. Compared to step therapy, stratified treatment was found to have a quicker effect on headache relief. As a result, stratified therapy is preferable over step-care therapy [14].

Abortive/acute and prophylactic/preventive are the treatment options. The former seeks to halt the evolution of a headache. In contrast, the latter aims to lessen attack frequency, enhance responsiveness to the intensity and duration of acute episodes, and reduce impairment [15,16]. Abortive treatment includes nonsteroidal anti-inflammatory drugs (NSAIDs) like ibuprofen, naproxen, diclofenac, aspirin, or acetaminophen [17]. Triptans (the first line in patients with allodynia), which include sumatriptan, zolmitriptan, eletriptan, rizatriptan, and almotriptan, with or without naproxen, are used for moderate to severe attacks. Therapy needs to be customized because, unlike NSAIDs, patients who do not respond well to one triptan may respond to another. NSAIDs and triptan work better together than either drug class by itself [18]. Antiemetics: metoclopramide, chlorpromazine, and prochlorperazine are used as adjunctive therapy. CGRP antagonists include rimegepant and ubrogepant. These drugs are considered in patients who do not respond to conventional treatment or those with coronary artery disease [19]. Selective serotonin 1F receptor agonist, e.g., lasmiditan, is used for the acute treatment of migraine in patients who cannot use triptans due to cardiovascular risks [20]. Ergots, which include ergotamine and dihydroergotamine, are parenterally used for medication overuse headache and status migrainosus and are effective as bridge therapy [21]. Dexamethasone can reduce the recurrence of early headaches [22]. Some non-pharmacological treatments such as transcutaneous supraorbital nerve stimulation can reduce the intensity [23]. Transcranial magnetic stimulation is proved effective as a second-line treatment [24,25]. Some other therapies include nonpainful remote electric neurostimulation [26], peripheral nerve blocking (occipital plexus and sphenopalatine ganglion) [27], preventive treatment agents including beta-blockers such as metoprolol and propranolol, especially in hypertensive and non-smoker patients. Antidepressants such as amitriptyline and venlafaxine, are especially used in patients with depression or anxiety disorders and insomnia. Anticonvulsants such as valproate acid and topiramate are used in epileptic patients. Calcium channel blockers, verapamil and flunarizine, are especially advised to women of childbearing age or patients with Raynaud's phenomenon. CGRP antagonists include erenumab, fremanezumab, and galcanezumab [28].

Chronic Migraine Treatment

Topiramate, onabotulinumtoxin A, and various neuromodulation therapy techniques are among the few effective treatments for chronic migraines that have been supported by evidence [29,30]. In a few small studies, other preventive medicines such as amitriptyline, valproate, gabapentin, and pregabalin have also demonstrated efficacy in treating chronic migraine [31]. Invasive and noninvasive neuro-modulatory techniques, such as greater occipital nerve blocking, occipital nerve stimulation, vagal nerve stimulation, and transcranial magnetic stimulation, may be helpful for patients with pharmacologically refractory chronic migraines [23-27].

Pathophysiology of migraine

**Figure 2:**
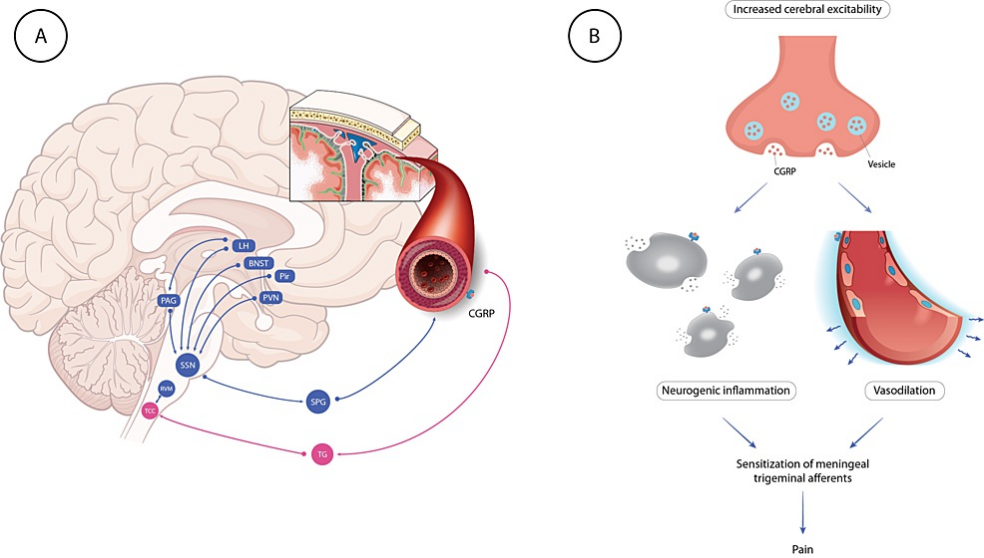
Pathogenesis of migraine and the role of CGRP BNST: bed nucleus of stria terminalis; LH; lateral hypothalamus; PAG; periaqueductal gray; Pir: piriform cortex; PVN: paraventricular hypothalamic nucleus; SPG: sphenopalatine ganglion; SSN: superior salivatory nucleus; TCC: trigeminal cervical complex; TG: trigeminal ganglion; RVM: rostral ventromedial medulla; CGRP: calcitonin gene related peptide Image Credit: Authors; parts of the illustration are made using content from smart.servier.com

Migraine has numerous phases, including interictal, premonitory, aura, headache, and postdrome. Over the past few years, our understanding of the etiology of each migraine phase has improved. Several cortical and subcortical brain regions, including the hypothalamus and brainstem nuclei that modulate nociceptive transmission, interact intricately during the premonitory phase, which can start up to three days before the period of headaches. The trigeminovascular pathway, a thoroughly studied route, is activated during the headache phase. One-third of patients may experience a cortical spreading depression-like event during an aura phase in some attacks, characterized by a gradually spreading pulse of glial and neuronal cell depolarization and hyperpolarization [32-35]. Various areas of the brain are involved in the pathogenesis of migraines [32]. Figure 2 shows a diagrammatic compilation of the same.

Phase 1: Premonitory - Homeostasis and Meningeal Nociceptors Alterations

Some people can predict the commencement of a migraine headache up to 12 hours in advance, thanks to the premonitory phase of the condition, which can start up to three days before a migraine headache [32]. Studies using functional neuroimaging demonstrate that the hypothalamus has a role in premonitory behavior. The posterolateral hypothalamus, midbrain tegmental area, periaqueductal gray, dorsal pons, and other cortical regions were all activated during the premonitory phase of a positron emission tomography (PET) study in individuals with glyceryl trinitrate-induced migraine attacks. Other pathways may have a role in migraines brought on by stress. It has been shown in preclinical models that sympathetic outflow into the meninges, which includes norepinephrine release, influences pronociceptive signaling by acting on dural afferents and dural fibroblasts. In reaction to the release of dynorphin and corticotropin-releasing hormone brought on by stress, which may also contribute to the development of stress-induced migraine, there's a chance the kappa-opioid system will work. These physiological mechanisms, which entail circuits that extend over to preganglionic parasympathetic nerves in the superior salivatory nucleus, might trigger the release of neurotransmitters from parasympathetic neurons that subserve the meninges and meningeal circulatory system, activating peripheral nociceptor cells. The current rhythmic cycle of cyclical brainstem activity may also influence whether the premonitory phase changes into the headache phase. When the rhythmic brainstem activity is strong, the cutoff for the conduction of nociceptive trigeminovascular impulses is raised, and nociceptive signals are repressed. Because low cyclical brainstem activity reduces the limit for the propagation of nociceptive signals, a migraine headache may manifest as a result [32-36].

Phase 2: Aura - Cortical Spreading Depression (CSD)

An aura precedes a migraine attack in about one-third of cases. According to ICHD-3, migraine with aura is characterized by recurrent episodes of unilateral, completely reversible, visual, sensory, or other CNS symptoms. These episodes start gradually and are followed by headaches and other migraine-related symptoms. The neurophysiological equivalent of migraine is supposed to be CSD, which Aristides Leo first observed in 1944. It can be recognized by a slow waveform of depolarization in the membranes of neuronal and glial cells that glides smoothly (2-6 mm/min); the beginning and progression of aura symptoms are accompanied by a drop in the brain activity that can last for up to 30 minutes. This wave of spreading depression is preceded by a wave of hyperemia, followed by a prolonged phase of cerebral oligemia. Localized increase in extracellular potassium (K+) lasting for 30-50 seconds causes chronic depolarization of neurons (CSD). One hypothesis states that the first extracellular K+ increase results from recurrent depolarizations and repolarizations of hyperexcitable neurons located in the cerebral cortex. The cells from which this extracellular K+ concentration was released are then further depolarized as a result. This considerable disruption of the ionic gradients in cell membranes, the inflow of sodium (Na+) and calcium (Ca2+), and glutamate release occur simultaneously with this enormous K+ outflow. There are many theories regarding CSD dissemination, but they are still poorly understood. Contrary to prior beliefs, which suggested that CSD is propagated by interstitial transport either of K+ or glutamate, more recent research suggests that gap junctions among glial cells or neurons regulate the spread of CSD. Animal studies have provided mounting evidence that CSD can trigger trigeminal nociception, and this in turn triggers headache pathways [36-40].

Phase 3: Headache - Trigeminovascular Pathway Activation And Central-peripheral Sensitization

Trigeminovascular pathway activation is assumed to be the primary cause of the typical throbbing pain of migraine headaches. The anatomy and physiology of the trigeminovascular system, which is widely known, explains how migraine pain is distributed. Nociceptive information is moved from the meninges to the brain's core regions and subsequently to the cortex by the trigeminovascular system. The trigeminal ganglion-derived nociceptive fibers innervate the meninges and main cerebral arteries. Vasoactive neuropeptides such as CGRP, and in response to stimulation of the nociceptive neurons that innervate the dura mater, pituitary adenylate cyclase-activating polypeptide-38 is produced. Although the exact degree of these effects is yet unknown, this signaling through the trigeminovascular route results in arterial dilatation, mast cell degranulation, and plasma extravasation. Some people believe that CSD causes the meningeal nociceptors to activate. Locally generated substances such adenosine triphosphate (ATP), glutamate, K+, h+, CGRP, and nitrous oxide are thought to diffuse to and trigger meningeal nociceptors. After being stimulated by endogenous mediators, peripheral trigeminovascular neurons become sensitized to dural stimuli, which lowers their threshold for response and increases the magnitude of their response. Peripheral sensitization is assumed to be the source of the migraine's characteristic throbbing pain and the reason why leaning over or coughing makes the agony worse. By sensitizing central trigeminovascular neurons in the trigeminocervical complex (TCC) and thalamic nuclei, cephalic and extracephalic allodynia is brought on. Sensitization increases the responsiveness to benign cephalic and extracephalic stimuli as well as spontaneous brain activity [41-45].

The Function of CGRP

There are various locations along the trigeminovascular circuit where CGRP is thought to act. Meningeal nociceptors may become activated and sterile inflammation may occur because of peripheral CGRP synthesis in the meninges, which also causes arterial vasodilation. In the trigeminal ganglion, CGRP has a central role in signaling between trigeminal ganglion neurons and a peripheral one in promoting plasma extravasation by boosting substance P secretion (although the importance of this activity in migraine is uncertain). In the trigeminal ganglion, neuronal-glial cell signaling has also been connected to CGRP, and this connection has the potential to influence peripheral sensitization. The TCC's ability to produce CGRP may increase the release of neurotransmitters from adjacent primary afferent terminals, facilitating nociceptive transmission [46-49].

Diagnostic approach

While the primary focus of this article remains on the clinical assessment and treatment of migraine, a brief description of other types of headaches remains essential, as other primary headache disorders can be misdiagnosed as migraine. Adding to the complexity, one or many primary headaches etiologies may coexist, usually migraine with cluster headaches [50]. Secondary headaches, as previously mentioned in the categorization of headaches, are symptoms of a sinister pathology and must always be ruled out when a patient arrives for the first time in a healthcare environment with an acute onset headache.

Secondary headaches must be suspected when one or more of the following points exist [51]: Deviation from previous headache pattern, headache onset after 50 years of age, sudden onset “worst headache of their life”, progressively worsening headaches, acute onset with occipito-nuchal location, focal neurological symptoms (except for aura), headaches that are accompanied by systemic symptoms such as fever, stiff neck, or rash, decreased level of consciousness, new onset in a patient with cancer or HIV, headache always on the same side (‘‘side-locked headaches’’), post-traumatic headache, headaches that awaken the patient from sleep, or headaches that do not meet primary headache criteria.

It is imperative to carefully examine for signs such as papilledema for increased intracranial pressure and nuchal rigidity for meningeal irritation. Tender nodular temporal arteries suggest giant cell arteritis [52]. Other primary headache disorders vary in their presentation and can be ruled out based on history and a clinical examination. Each of these differs from migraine in their presentation. Tension-type headaches present as a tight band around the head, bilateral, pressing, and dull in nature, Photophobia and phonophobia are occasionally present. Unlike migraine headaches, nausea and vomiting are classically absent [53]. A cluster headache is a trigeminal autonomic, cephalalgia, unilateral headache, presenting as orbital, supraorbital, temporal, or a combination lasting 15 minutes to three hours and occurring eight times per day. Attacks have a remission period of months to years, and a single attack usually lasts from a few weeks to months. Same-sided autonomic manifestations accompanied by a sense of restlessness or agitation, or both, are also seen [54,55].

**Figure 3:**
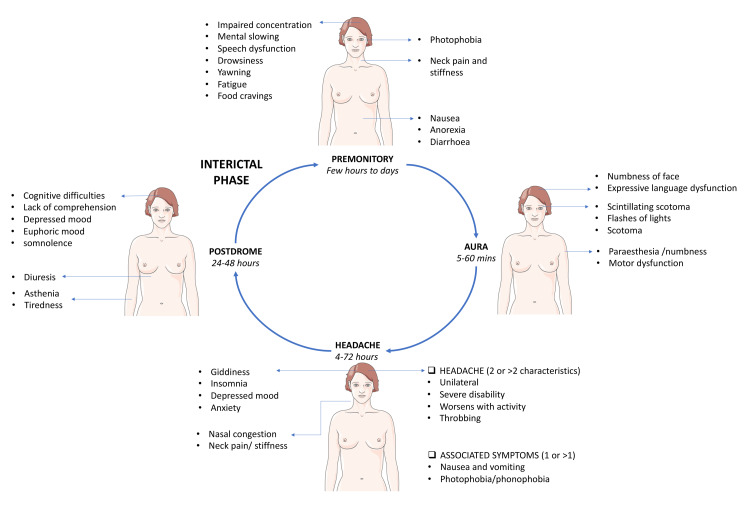
Various phases of migraine and the associated symptoms Image Credit: Authors

Migraine is a clinical diagnosis. With the presence of consistent history and normal physical examination, no laboratory studies are needed to make the diagnosis [52]. Discussing symptomatology and its significance in ascertaining the diagnosis remains vital. With a widespread prevalence of about 18% in females and 6% in males, only half of the migraine sufferers have their condition diagnosed, and about a third are receiving treatment [56]. Migraine is a disabling headache having social, occupational, and familial consequences. It is imperative to evaluate the effect it has on the quality of life in addition to the diagnosis [57]. Assessing the discomfort and limitations brought by headaches will help the primary care provider customize the treatment [58]. A thorough history including onset, duration, site, lateralization, intensity, evolution, pulsating or persistent pain (exacerbating and mitigating factors), provoking factors, premonitory symptoms, aura along with its properties such as nature, duration, temporal relation to headache, and assessment of associated symptoms like nausea, vomiting, photophobia, and phonophobia helps in the diagnosis [57]. Aura is defined as a reversible, gradually developing focal neurological symptom, often described as a binocular vision, but may also present as unilateral sensory, motor, or speech disturbance. It lasts about 5-20 minutes, usually not over one hour. Sensory aura is described as a pins and needles sensation on half side of the body [59]. Commonly described types of visual aura include a positive or a negative visual aura or a preceptory visual disturbance. Positive aura is a “march” of zigzag lines arranged in semicircular shape but can also be experienced as white specks, tinted spots, and lines that can be bent or straight [60]. Speech disability can range from moderate linguistic difficulty to complete inability to speak [61]. Arteriovenous malformations [62], carotid artery dissection [63], epilepsy [64], subarachnoid bleed [65], and occipital lobe neoplasm [66] may also present with predominant visual symptoms like migraine, associated with headache, and, hence, form close differentials for migrainous aura. Figure 3 depicts the various phases of migraine and its associated symptoms.

A very concerning differential diagnosis of a migrainous aura is a transient ischemic attack (TIA), as both have similar presenting complaints. Visual manifestations of TIA are characterized by their brevity of onset and duration but differ in the description as “a curtain pulled over the eye” [67]. Headaches meeting the criteria of two pain symptoms and one associated symptom are classified as migraine headaches. It is noteworthy to mention that exceptions exist, as migraine headaches can be bilateral or non-throbbing. But one thing that remains constant is that the patient is clinically normal in the inter-attack period with an unremarkable neurological examination. The ICHD-3 code is then used to classify migraines. Traditionally, migraine is classified by the International Headache Society (IHS) system, but it has proved inconvenient to apply by primary care physicians at the point of healthcare contact [68]. The introduction of a three-item identification of migraine or the ID-Migraine™ tool addresses this concern. It is a patient-administered scale. For the scale to apply to a patient, the following two criteria must be fulfilled prior: (i) A record of two headaches or more in the past three months and (ii) The patient must be willing to speak about their headache to their healthcare provider, or they must have had a headache that impairs quality of life, in a sense, hampers their ability to work, enjoy, or study. The scale includes photophobia, nausea, and disability. Any two out of three criteria hint towards the diagnosis of migraine [69,70].

**Figure 4:**
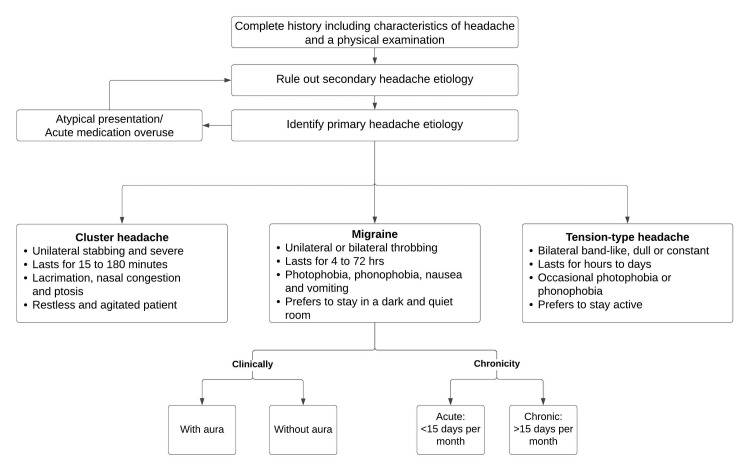
Approach to diagnosis of migraine

The Migraine Disability Assessment (MIDAS) questionnaire provides a yardstick to interpret the disability due to migraine headaches. It encompasses both lost days due to disability and the actually missed workdays, describing the disease's impact. MIDAS, a five-item questionnaire, has been tailored for migraine disability evaluation [71]. The MIDAS grading divides patients into four disability categories: (i) none to little, (ii) mild, (iii) moderate, and (iv) severe. MIDAS is intended to capture the cumulative impact of illness over a specific period [71,72]. Besides disability evaluation, MIDAS is used to stratify the management of migraine patients [73]. In most cases of migraine, investigations are not required [52]. However, if a patient is referred to a neurologist or emergency room, they will undergo a detailed clinical examination in addition to diagnostic studies [74]. Blood samples taken are analyzed for infectious, inflammatory, metabolic, or hormonal pathologies [75]. Imaging modalities like MRI and CT scan might be ordered in an atypical pattern of symptoms to probe for intracranial pathologies like brain tumors [74]. Specialized testing modalities are individualized according to the patient, including lumbar puncture, electroencephalography, cerebral arteriography, magnetic resonance angiography, spiral CT angiography, carotid artery or heart ultrasound, transcranial Doppler, and echocardiogram [75]. A simplified summary of the approach to diagnosis is described in Figure 4 [50-74].

Prophylaxis

FDA-approved prophylactic agents are divalproex sodium, propranolol, topiramate, and timolol. Prophylaxis treatment requires continued daily administration of drugs irrespective of migraine attacks [76]. It is initially started in patients with four or more headaches per month and should be continued for at least two to three months daily [77,78]. The major classes of drugs used in the prophylaxis of migraine are listed in Table 2. 

**Table 2:** Classes of drugs used in prophylaxis of migraine CGRP: calcitonin-gene related peptide

Class	Drug
Herbal medications	Petasites, feverfew, Zhengtian capsule
Nutritional supplements	Vitamin D, riboflavin, magnesium, coenzyme Q10
Antiepileptics	Divalproex sodium, topiramate
Antidepressant	Amitriptyline, venlafaxine
Beta-blockers	Propranolol, timolol
Calcium channel blockers	Verapamil, flunarizine
Neurotoxins	Onabotulinum toxin A
CGRP monoclonal antibodies	Erenumab, fremanezumab, eptinezumab, galcanezumab

Prophylaxis in Pregnant Patients

Prophylaxis is prompted when patients have more than three to four severe attacks per month. However, the potential risk to the fetus caused by the prophylactic drugs should be explained to pregnant patients. Propranolol (10-20 mg) twice daily is reported to cause fetal dehydration, distress, and growth retardation. It is reported to cause reversible fetal or neonatal bradycardia [79-89].

Prophylaxis Options

We recommend individualizing the therapy for each patient and choosing the best fit for each patient, instead of one-size-fits-all. The best choice can be made from the available options.

Herbal Options: Petasite extract 75 mg twice a day is a well-tolerated and effective herbal medication without apparent hepatotoxic side effects at this dose and is used for decreasing the frequency of migraine attacks. It is an effective option to be referred to in adults [79-81]. It is also well tolerated and effective in children and teenagers at lower doses [82]. Further monitoring of the liver profile is important due to its metabolic effect on the liver when taken for a longer duration at a higher dose [83]. Feverfew (*Tanacetum parthenium*) has shown better results than a placebo in a few studies when given thrice daily in a patient with mild migraine at a dose of 6.25 mg [84,85]. Zhengtian capsules comprising 15 different traditional Chinese herbal medications showed efficacy comparable to flunarizine [86,87].

Extended Cycle Combined Oral Contraceptive Pills: Menstrual migraine can be prevented using cyclic dosing of extended cycle combined oral contraceptive pills along with frovatriptan, zolmitriptan, and naratriptan during hormone-free intervals, but is associated with new symptoms of headache once withdrawn [20,36].

Tricyclic Antidepressant: Amitriptyline 10-150 mg is used prophylactically. A very low dose (10 mg) or low dose (10-25 mg) daily at bedtime is the initial preferred dose; for patients with comorbid depression, a higher dose is preferred [90,91]. It is well tolerated at low doses, and the common adverse effects observed in patients are daytime fatigue and dry mouth [90]. Other adverse effects include dizziness, weight gain, somnolence, and sinusitis [92].

Topiramate: It is given 100 mg/day orally after slowly increasing the dose from the initial 25 mg/day [26,56]. Topiramate is the first line of treatment for migraine prevention in patients with coexisting complaints of weight gain epilepsy [93]. In pediatric patients, caution must be exercised when using topiramate for prophylaxis, even at a low dose (0.9 mg/kg/day). It is associated with poor vocabulary development without affecting speaking [94]. Nephrolithiasis is a common side effect when topiramate is used for extended periods [95]. Other adverse effects routinely observed in patients are nausea, paresthesia, weight loss, somnolence, and hypoesthesia [92].

**Figure 5:**
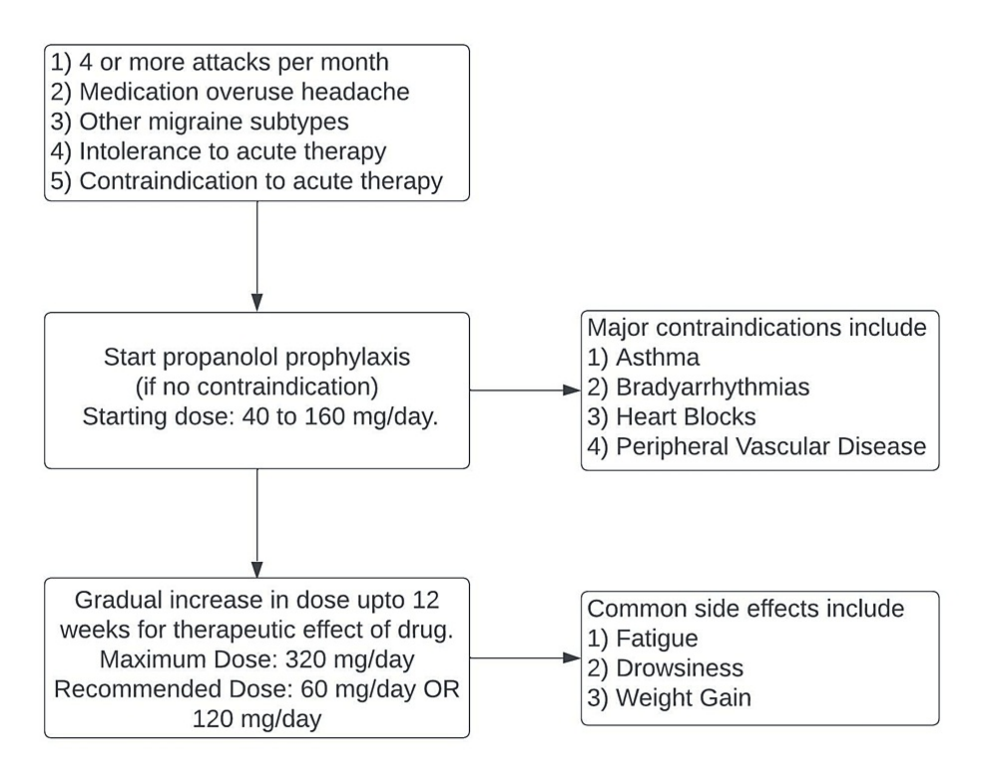
Beta-blockers and prophylaxis of migraine

Beta-blockers: Timolol and propranolol are two FDA-approved beta blockers used for migraine prophylaxis. Propranolol is the most commonly used drug for migraine prophylaxis (Figure 5). It can be considered in patients with hypertension [96]. Propranolol is associated with weight gain, unlike timolol, which is weight neutral [97]. 

Nutritional Supplements in Prophylaxis: Nutritional supplements have insufficient evidence to reduce the duration and frequency of migraine attacks to be used as monotherapy. But it can be added to courses based on other prophylactic agents. Some studies show that vitamin D, riboflavin (400 mg OD), Co Q10, Magnesium (200 mg twice daily), and omega-3 fatty acids are cost-effective and beneficial add-ons [30]. Other studies in pediatric patients have shown the benefit of conventional prophylactic agents over nutritional supplements [98,99].

Calcium Channel Blockers: Flunarizine is a calcium channel blocker; its mechanism of action in migraine is unknown. One proposed mechanism is related to monoamine uptake and metabolism when used in migraine [100]. Its anti-hypoxic and anti-vasoconstrictive effects are also considered [101,102]. It is used in vestibular migraine due to its effect on vertiginous symptoms [96]. Patients taking 10 mg tablets before sleep for 12 weeks showed an improvement compared to a placebo in a meta-analysis. It has also shown a decrease in the frequency and duration of migraine attacks in patients with classical migraine [103-105]. It is also seen that flunarizine is a better choice as it has a better safety profile than propranolol. Propranolol causes various cardiac side effects, including a decrease in blood pressure and heart rate, which are not seen with the use of flunarizine [101]. Sedation (somnolence) and weight gain are side effects seen in patients using flunarizine [100,106]. Studies show that the use of flunarizine is associated with an increase in leptin levels, causing an increase in BMI. This is also the cause of weight gain due to amitriptyline [107]. It can be combined with topiramate as they have opposite actions on weight [108]. Few studies also showed an increase in the 21-item Hamilton rating scale for depression when used in comparison to valproate [109]. It is also an effective drug for prophylaxis in childhood migraine. It is given 5 mg orally before sleep and labeled as the drug of choice to treat the pediatric population. A decrease in symptoms was observed in patients within two to four weeks after the initiation of prophylaxis [110-112].

Yoga and Aerobic Exercise: In patients with migraine, a disturbed routine is associated with an increased risk of migraine attack onset. Regular routine life with aerobic exercises and various muscle relaxation techniques, combined with prophylactic agents, shows better results [113,114]. Systematic reviews and meta-analyses have shown that yoga helps treat tension-type headaches while studies haven’t shown any effect in patients with migraine [115].

Divalproex Sodium: Divalproex sodium is recommended in low doses for prophylaxis (500 mg/day). It significantly reduces migraine episodes' severity, frequency, and duration [116,117]. A few studies also showed that concurrent use of magnesium with divalproex sodium for prophylaxis results better than non-concurrent use [118]. Drugs like levetiracetam are less effective but can be used if valproate is contraindicated as it is well tolerated [119,120]. Studies also showed that drugs like cinnarizine are more effective and without any significant side effects compared to valproate [121].

CGRP Monoclonal Antibodies and Gepants: The FDA has approved four CGRP monoclonal antibodies to prevent migraine: erenumab, fremanezumab, galcanezumab, and eptinezumab. Goadsby and colleagues conducted a randomized control trial (RCT) of over 900 patients and concluded that erenumab 70 mg and erenumab 140 mg had a superior outcome compared to a placebo in terms of 50% or more reduction of mean migraine days per month and number of days acute migraine-specific medication was used. However, they did not comment on the long-term efficacy and safety of the drugs [122]. Reuter and colleagues conducted another RCT in a pool of patients who had previous two to four previous unsuccessful preventive trials and concluded that erenumab was superior to a placebo with a 50% or greater reduction from baseline in the mean number of monthly migraine days at 12 weeks [123]. The recommended dosage of erenumab is 70 mg/140 mg s.c. once monthly and these injections are administered in the thigh or the belly and can be self-injected [122,123]. Patients on erenumab should be monitored for the worsening of hypertension [124].

Eptinezumab is given as a 100 mg IV infusion over ~30 minutes every three months [125]. The Prevention of Migraine via Intravenous ALD403 Safety and Efficacy-1 (PROMISE-1) trial demonstrated that 100 mg and 300 mg of this drug significantly reduced the mean monthly migraine days compared to a placebo. Also, the reduction in migraine days was >50% in more patients taking the same doses than those treated with placebo [126]. Winner et al. identified that treating patients experiencing moderate to severe migraine attacks with intravenous eptinezumab achieved quicker symptom resolution than placebo [127].

Fremanezumab, also known as TEV-48125, is a fully humanized immunoglobulin G2a monoclonal antibody that binds to CGRP and prevents it from binding to receptors [128]. In the fremanezumab-treated groups, the treatment was related to an improved number of headache-free days and normal functional performance on all measures for patients with episodic migraine and some measures for individuals with chronic migraine [129]. According to a recent study by Ferrari et al., it is efficacious and well-tolerated in individuals with difficult-to-treat migraine who had previously failed to respond to up to four classes of migraine-preventative medicines. Furthermore, fremanezumab appears to be more beneficial in real-world settings than in RCTs. Younger age appears to be a possible predictor of response. The recommended dose of fremanezumab is 225 mg once monthly or 675 mg (three consecutive injections of 225 mg each) every three months administered subcutaneously [130].

CGRP is an appealing prospect for preventing migraines. Inhibition of the CGRP pathway is a physiologically specific, disease-targeted strategy for migraine prevention that significantly improves the therapy of a prevalent and debilitating neurologic disease [131]. Galcanezumab, a humanized monoclonal antibody that binds to CGRP and inhibits its physiological activity, has evolved as a potent option [132]. Patients with episodic migraine who were treated with galcanezumab showed a significantly higher reduction in monthly migraine headache days and better compliance compared to those who were treated with placebo [133-135]. The recommended galcanezumab loading dose is 240 mg, followed by monthly doses of 120 mg subcutaneously [135].

Acute therapy of migraine

Migraine is a common and functionally disabling neurological condition characterized by unilateral recurrent pulsatile headaches lasting for 4-72 hours and often associated with symptoms including nausea, vomiting, and allodynia. Migraine accounts for a significant financial burden due to increased medication costs used for treatment as well as for mental disorders like anxiety, depression, epilepsy, and stroke-like conditions to which a person is prone due to migraine. Acute and preventive therapies can help to reduce the headache, severity, and other symptoms of this disease, thereby restoring normal functions [136]. Migraine has been subdivided into two categories by the international headache society: migraine with aura and migraine without aura (Table 3) [8]. Acute treatment is given to relieve a migraine attack. The treatment aims to free individuals from pain and associated symptoms with little or no adverse effects and restore functional capacity with minimal need for repeated dosing or rescue medications [137]. All migraine sufferers should be considered for a trial of pharmacological and non-pharmacological treatment strategies. Managing migraine includes many aspects involving lifestyle modifications like regular exercise, nutrition, stress management, and medications. Journaling migraine attacks and the above management can help plan individualized treatment for acute migraine [138]. Drugs used in acute treatment should be individualized according to patient needs, primarily depending on pain severity, recurrence rates, associated symptoms, and relevant medical history [139].

**Table 3:** International Headache Society categories for migraine

Migraine without Aura	Migraine with Aura
Minimum attacks alongside criteria mentioned under	Minimum attacks alongside criteria mentioned under
Migraine lasts for 4-72 hours (untreated or successfully treated)	Migraine without aura begins during aura or follows aura within 60 minutes
Headache has at least two characteristics: (i) Unilateral location; (ii) Pulsating quality moderate to severe pain intensity, (iii) Aggravation by or causing avoidance of routine physical activity (e.g., walking or climbing stairs)	Aura presents with at least two features: (i) Fully reversible visual symptoms both positive (flickering spots, lights) and negative (loss of vision); (ii) Fully reversible sensory symptoms both positive (pins, needles) and negative (numbness); (iii) Fully reversible dysphasic speech disturbance
In additionally to headache, at least one of the following is seen: (i) Nausea or vomiting, (ii) Photophobia or phonophobia; (iii) No association with other diseases	At least two of the following: (i) Homonymous visual symptoms and/or unilateral sensory symptoms; (ii) At least one aura symptom develops gradually over >5 minutes and /or different aura symptoms occur in succession over >5 minutes; (iii) Each symptom lasts >5 and last for <60 minutes; (iv) No association with other diseases

Drug Approvals for the Treatment of Acute Migraine Treatment

Drugs used for migraine are from two classes: (i) Specific antimigraine drugs, like 5-HT agonists (ergots, diptan, triptans) and anti-CGRP-acting drugs (gepants), and (ii) General analgesics (COX inhibitors), commonly taken to relieve pain. The FDA, between 1970 and 2020, has approved 17 drugs for migraine [140].

Ergot Alkaloids: Dihydroergotamine (DHE) is a serotonin receptor agonist acting through 5-HT1B and 5-HT1D receptors. The former receptors constrict the intracranial extracerebral blood vessels, whereas the latter inhibits trigeminal neurotransmission [141,142]. Before triptans, ergots and DHE were widely used as specific antimigraine drugs based on the presumption that migraine occurs due to increased sympathetic stimulation and ergots inhibit the pressor effects of adrenaline [143]. These drugs are of prime importance for attacks lasting > 48 hours, they are also useful in reducing the recurrence rate of migraine attacks [144]. DHE can be administered by various routes: IV injection, intranasal delivery, and intramuscular/subcutaneous injection. Oral DHE is less common, due to poor absorption and extensive first-pass metabolism. While the intranasal route is noninvasive and rapid due to the large surface area for absorption, use is restricted due to the dispersion of aerosols causing reduced action on lungs and oropharyngeal deposition. A greater incidence of side effects like nausea, dizziness, paresthesia, and reduced gastrointestinal motility was evident following IV dosing [145]. On the standards of efficacy and tolerability to nausea, recommended dose of 0.5-2 mg dose of DHE per attack should be administered as early as possible. The adverse effects of ergot usage are ergotamine overuse headaches and the relatively rare overt ergotism.

Triptans:* *Triptans have ergot-like properties (serotonin 5-HT1B/1D receptor agonist activity) and are used in moderate to severe migraine. Studies claim triptans to be superior to placebo in short, sustained headaches [146]. Standard dose triptans cause headache relief in 42-76% of patients within two hours as compared to 27% with placebo treatment. The percentage of rescue medications used by patients ranged from 20% to 34%, as compared to 52% for placebo [147]. Seven different triptans are currently marketed [140] of which sumatriptan, zolmitriptan, rizatriptan, naratriptan, almotriptan, and frovatriptan bind to both 5-HT1B and 5-HT1D receptors whereas eletriptan binds to 5-HT1B, 5-HT1D, and 5-HT1F receptors. Triptans, although having relatively similar molecular structures, differ in pharmacokinetics, which accounts for varying efficacy and tolerability [139]. In a study, eletriptan tablet (21%) and zolmitriptan tablet (24%) were found to be requiring the least amount of rescue medications as single drugs, whereas NSAIDs (37%), sumatriptan (34%), and acetylsalicylic acid (ASA) (33%) used the most. Comparatively, rizatriptan and eletriptan are found to be more effective in pain relief than other triptans. The pain and headache comfort at two hours for eletriptan was around 67.7% and 71.6%, respectively, and 24-hour pain and headache response was 54.1% and 87.8%, respectively. The second-best and third-best were rizatriptan and zolmitriptan on the list of triptans. Triptans are sold with varying strengths and formulations, like oral, orally disintegrating, nasal sprays, and subcutaneous injections [148]. The above findings are in accordance with the “common” doses of triptans. Sumatriptan is commonly used in a 100 mg dosage. Treatment dosage and choice of the drug are of prime importance considering disease outcome. Sumatriptan (SC 6 mg) has the lowest number needed to treat (NNT) than other triptans [149]. Triptans yield the best results when taken early. For migraine with aura, taking triptans at the onset of pain rather than the onset of the aura has been thought to be best, although taking triptans during a typical aura seems safe. Triptans have relatively lesser adverse effects due to vasoconstriction as compared to ergots but are contraindicated in patients with cardiovascular/cerebrovascular anomalies and hemiplegic migraine [150].

NSAIDs: These are commonly accessible as over-the-counter drugs for migraine treatment. Being COX 1 and 2 inhibitors, they inhibit neurogenic inflammation and cause a reversal of central sensitization in migraine [151]. Different NSAIDs like ibuprofen, naproxen sodium, ASA, diclofenac potassium, ketorolac, etc. are used for acute treatment. Ibuprofen (400 mg) is commonly consumed; it is a non-selective COX inhibitor having a rapid onset of action, and NNT for pain response at two-hour intervals is 3.2 [152]. The use of low-dose ibuprofen has also been effective in reducing symptoms of photophobia and phonophobia associated with migraine [153]. Diclofenac potassium is a rapidly acting drug with < 15 minutes of action with the oral route; the commonly used dosage is 50 mg and its NNT is 8.2 for pain relief at two hours. Its adverse effects are mild and considerate to placebo [154]. Naproxen (500 mg) is a longer-acting drug and is useful in moderate to severe migraines. The adverse effects of dizziness, dyspepsia, and abdominal pain are more as compared to placebo [155] but compared to other NSAIDs, it has lesser cardiovascular side effects and, hence, benefits patients with cardiovascular comorbidities [156]. Aspirin (1000 mg) has an efficacy comparable to that of sumatriptan and is widely used in moderate to very severe migraine attacks. The addition of antiemetics (metoclopramide 10mg) may reduce symptoms of nausea and vomiting in migraine [157].

Acetaminophen: An effective acute migraine treatment strategy. Metoclopramide (10 mg) combined with acetaminophen raises the short-term effectiveness nearly to oral sumatriptan (100 mg). The standard dose for pain relief amounts to 650-1,000 mg. A patient generally needs repeated dosing for a long-lasting symptom-free period as it has a shorter half-life (two to three hours) and optimum plasma concentrations are reached within 30-60 minutes [158]. Acetaminophen acts centrally and inhibits prostaglandin synthesis in neurons. As acetaminophen doesn’t inhibit the COX system but rather reduces COX activity, it is favorable and causes relatively fewer gastrointestinal side effects than NSAIDs. Also, it does not alter platelet function due to its central action thence prostaglandin synthesis in platelets is unaffected [159].

Treatment strategies

The treatment of migraine is multidimensional and requires intervention at different phases of the disease. Treatment involves the management of the acute episode followed by a plan to prevent recurrent episodes. A specialized approach may be mandated for chronic migraine. Further, special populations, such as pregnant patients, require special consideration.

Treatment strategies are generalized based on severity. For mild to moderate intensity headaches, acetaminophen or a NSAID is started. This is the most standard unless the patient comes after taking over-the-counter medications. In case of failure, triptan medications should be started. For those with severe disabling attacks, triptans are given immediately if there are no contraindications. Some patients may require more than one strategy at various times depending upon attack severity. For refractory migraine attacks, various rescue medications may be necessary. Migraine strategies in relation to patients can be divided into the following: stratified, step-care-across-attacks, and step-care-within-attacks [154]. In "stratified care," the medication chosen for a patient is based on attack severity and/or degree of migraine-related disability. In "step care within an attack," a simple analgesic or NSAID is used initially for a migraine attack. If the first medication is not successful, another medication (e.g., a triptan), is used. In “step care across attacks," the physician prescribes an initial medication (e.g., NSAID), which is followed for further attacks. If found ineffective, it is replaced by other higher effective ones (e.g., a triptan.)

In general, many patients with migraine would have tried several non-prescription medications before consulting the doctor; therefore, a “step care across attacks” is already present where a physician prescribes a more effective medication. “Stratified care” is likely to be the most effective and cost considerate treatment approach and, hence, used in most severe migraine attacks. “Step care within an attack,” although accepted, has the disadvantage that failure of the first medication may lead to unresponsiveness to the more effective medication taken because it may be no longer effective as it would have been if taken earlier [14].

Medication-Overuse Headache

Medication-overuse headache takes into account headaches lasting more than 15 days per month in previously diagnosed migraine patients and taking regular medications for 10 or more days for more than three months [8]. Patients should be consulted for cautious use of antimigraine medications [139]. Depending on the type of medication, it can be classified into triptan, analgesic, opioid, or combined medication overuse [140]. The use of opioids and combined medications are generally avoided as first-line treatment as they are less effective and increase the risk for medication-overuse headache. Patients with migraine requiring drugs constantly should be advised to restrain medication use to nearly two headache days per week. Patients who can’t limit usage are given preventive therapy to prevent medication-overuse headache and headache severity [136]. Changes in acute treatment medications can also help in restricting medicine usage. Repeated use of some newer drugs like CGRP receptor agonists gepants (ubrogepant and rimegepant) doesn’t increase the risk of medication-overuse headache [160,161]. Neuromodulation therapy can reduce acute medication use [162].

Emerging treatment and special scenarios

FDA Approved But in Continued Clinical Trials

Lasmiditan: It is a ditan belonging to a novel drug class that targets the 5-HT1F receptor for the treatment of acute migraine. It’s a highly selective 5-HT1F agonist and was approved in 2019. Ditans are structurally different from triptans. Triptans bind non-selectively to 5-HT1B and 5-HT1D receptors and with varying affinity to 5-HT1F receptors, causing direct vascular vasoconstriction. In contrast, ditans are selective for the 5-HT1F receptor, and their mechanism of action is neuronal, without evidence of vasoactive effects [163]. According to preclinical research, lasmiditan binds to 5-HT1F receptors to decrease CGRP output [164]. For patients with cardiovascular risk factors, triptan contraindications, or unpleasant side effects, lasmiditan is a promising acute antimigraine medication. However, lasmiditan is linked to CNS-related adverse effects such as drowsiness, paresthesia, and dizziness [165]. A phase-3 experiment is now evaluating the safety and effectiveness of lasmiditan for the treatment of acute migraine in children.

Rizatriptan: Triptan was licensed by the FDA in 1998 and is currently under several clinical trials, including a phase-2 clinical trial for the treatment of episodic dizziness in vestibular migraine and phase-3 research for the acute treatment of migraine in conjunction with naproxen or meloxicam.

Rimegepant: FDA approved it in February 2020. When administered orally, rimegepant takes two hours to reach its peak plasma concentration. Rimegepant has a modest side effect profile that is comparable to that of a placebo [166,167], with nausea and urinary tract infections being the most frequent occurrences. It can be used as a first-line anti-migraine medication in patients at elevated risk for cardiovascular events or in those with known cardiovascular illness because it does not constrict cranial vessels [168]. Additionally, it can be utilized as a backup strategy when triptans haven't worked [169].

Eptinezumab (Humanized IgG1 CGRP Ligand Antagonist) and Erenumab (IgG2 CGRP Receptor Blocker): Drugs that have already been licensed for migraine prevention are currently being tested for other indications. Examples include two antibodies, one targeting the CGRP receptor called erenumab (used to treat episodic and chronic migraine in children) and the other called eptinezumab used against CGRP itself (treatment-resistant patients with migraine and acute migraine). A total of 577 adults with episodic migraines were included in the 12-week, placebo-controlled, randomized, double-blind phase-3 ARISE trial to examine the efficacy of erenumab as a migraine preventative. The change of 58 in the average monthly migraine days (MMDs) was the primary goal. With 29.5% in the placebo group and 39.7% in the 70-mg group, there was a statistically significant difference in the proportion of persons with at least a 50% reduction of MMD (P =.01). Upper respiratory tract infection, discomfort at the injection site, and nasopharyngitis were the most frequently reported adverse medication reactions and were equivalent to placebo [170].

Erenumab was given to 955 participants in the STRIVE (Study to Evaluate the Efficacy and Safety of Erenumab in Migraine Prevention) trial at doses of 70 mg, 140 mg, or placebo to test its efficacy in preventing episodic migraines. The primary outcome was the shift in the mean MMDs from baseline to months four through six. The primary endpoint showed a significant difference, with the 70-mg group experiencing a decrease of 3.2 days, the 140-mg group experiencing a reduction of 3.7 days, and the placebo group experiencing a reduction of 1.8 days (P.001 for both groups when compared with the placebo). The erenumab groups and placebo groups both had comparable rates of adverse medication events [122].

Erenumab was the first FDA-approved CGRP antagonist for migraine prophylaxis in adults on May 17, 2018. The PROMISE-1 trial was a double-blind, randomized, parallel-group study that compares eptinezumab to placebo to see how well it prevents frequent episodic migraines. The frequency change of migraine days during a period of 12 weeks was the main objective. The average number of migraine days was considerably reduced by eptinezumab 30 mg, 100 mg, and 300 mg when compared to a placebo [126]. Additionally, phase-3 open-label research is being conducted to examine the security of using eptinezumab in repeated doses to treat chronic migraines.

Drugs in Clinical Trials

Amiloride:Presently, participants are being enrolled in phase-2 clinical research to assess its effectiveness in the prevention of adult migraine with aura. Acid-sensing ion channels (ASICs), a group of ion channels related to degenerin and epithelial sodium channels, are preferentially antagonistic to amiloride. Nerve growth factor (NGF) and 5-HT both affect ASICs, and 5-HT has recently been discovered to potentiate ASIC-3 via a non-proton ligand-binding site [171]. ASIC currents are also amplified by nitric oxide (NO) donors, one of the most effective migraine triggers in humans. Rats' trigeminal nociceptive responses are inhibited by blocking ASIC-3 in response to durovascular elicitation and NO-induced sensitization [172]. However, there isn't any concrete proof that amiloride works well for treating migraines.

Candesartan:Candesartan is now being studied in clinical studies for its efficacy and tolerance in individuals suffering from episodic and chronic migraine. Originally developed as an antihypertensive drug to stop the effects of angiotensin II, candesartan is an angiotensin II AT1 receptor antagonist. It is possible that either, both, or neither of the following processes contribute to the mechanism of candesartan migraine prophylaxis: (i) inhibition of excessive vasoconstriction brought on by 5-HT release, and (ii) inhibition of neurogenic inflammation. By preventing the activation of the AT1 receptor in the vascular smooth muscles, candesartan decreases vasoconstriction. Additionally, new studies show that candesartan pretreatment controls the development of vascular permeability. These results imply that candesartan may reduce migraine-causing neurogenic inflammation in the cerebral vasculature [173].

IONIS-PKKRx:According to a theory, bradykinin may play a role in the onset of vascular headaches by converting endogenous brain kininogens to bradykinin, which leads to bradykinin-dependent enlargement of the cerebral arteries and bradykinin-dependent stimulation of pain fibers [174]. When KLKB1 (plasma kallikrein) is targeted by the antisense oligonucleotide IONIS-PKKRx, the production of bradykinin is reduced. Recently, IONIS-PKKRx completed phase-2 clinical research investigating its safety, tolerability, and improvements in the frequency of migraine and headache days in adult patients.

Flunarizine: This is a 5-HT receptor antagonist and antihistamine (H1 receptor antagonist) acting on non-selective calcium ion channels and dopamine receptor antagonist [175]. Louis reported that taking flunarizine significantly reduced the number of migraine attacks compared to taking a placebo [176]. More double-blind placebo-controlled studies were conducted [177], because of this investigation. Flunarizine is now being evaluated in a phase-4 trial to see whether α-lipoic acid is effective as an additional medication for the treatment of adolescent migraine.

Lidocaine:The obstruction of the greater occipital nerve (GON) significantly affects the trigeminovascular system, which is crucial in the pathophysiology of migraines. A rat study found that mustard oil improved the excitability of meningeal afferent input by stimulating the GON and cutaneous C-fiber afferents. According to the study, there is a functional link between cervical afferents and nociceptive meningeal afferents in the GON [178]. It has been investigated whether lidocaine can be used to treat both acute and chronic migraines. The blockade of the sphenopalatine ganglion and the GON using a local anesthetic alone or in conjunction with a steroid-local anesthetic combination [179] have been proposed as promising targets for the effective treatment of acute migraine [180], despite the limited number of controlled studies that have been conducted.

Ketorolac: This is a nonspecific COX inhibitor. Trials evaluating the effectiveness of intravenous and intranasal ketorolac administration in pediatric migraine are currently being conducted. Individuals with migraines can benefit from oral, intravenous, and, more recently, nasal spray formulations since they absorb more quickly than oral medications and can be used in patients who experience nausea [181].

Auricular Puncture and its Effects on the Treatment of Migraine

Semi-permeable needles are inserted at precise locations (needle contact test); for example, the internal part of the anterior side of the antitragus, anterior part of the lobe, and upper concha. These points are proven to reduce migraine pains lasting up to 24 hours [182]. The location where the needle is to be inserted is assessed by pain grading on visual analog scale (VAS) [183]. For unilateral headaches, puncture on the same side is better, whereas bilateral needles are used for generalized pain. The intensity of headaches was much reduced in patients under acupuncture compared to those undergoing prophylactic therapy after four hours of treatment. Also, the side effects were lesser than those undergoing prophylactic two hours after treatment [184,185]. The duration of puncture therapies must consist of at least six intervals, two each week, although it may vary and not be defined [184].

Neuromodulation Therapy

Neuromodulation devices are useful in patients with relative difficulty in tolerating current therapeutics and who wish to have other non-pharmacological treatments. It also reduces acute medication overuse. FDA has accepted three devices for the acute treatment of migraine: (i) single-pulse transcranial magnetic stimulation (sTMS), (ii) the supraorbital transcutaneous nerve stimulator (STNS), and (iii) the noninvasive vagus nerve stimulator (nVNS) [186]. Usage of the devices is recommended for up to three months or longer for best results. This therapy highly indicated for patients suffering from chronic migraine episodes.

sTMS: sTMS is used for acute migraine prevention and different types of migraine. It works by changing the excitability pattern of cerebral cortex neurons, which induces pain. sTMS device produces a higher degree of pain relief at two hours (32/82 subjects, 39%) than those randomized to the sham device (18/82 subjects, 22%), with a therapeutic gain of 17% (95%CI 3-31%, P = .0179). The eNeura SpringTMS Post-Market Observational U.S. Study of Migraine (ESPOUSE) states that patients generally presenting with 9.06 migraine days in a month and using acute medications approximately 5.24 days/month had 2.75 lesser days of headache (P < .0001) and 2.93 fewer days of acute medication (P < .0001) after sTMS at month three. The device is safe to use with lesser side effects, the most prominent being lightheadedness, tingling, and tinnitus [187].

STNS: This is used for the prevention of episodic migraine, chronic migraine, and acute treatment. The main mechanisms involved are stimulating the supraorbital nerve and controlling pain transmission through the trigeminovascular system. Studies indicate STNS reduces migraine days per month to 6.94-4.88 days, with the P = 0.023 [187].

nVNS: nVNS mediates through vagal stimulation, affecting pain modulation, inflammation, and CNS excitability [187]. nVNS inhibits the brain structures producing neurotransmitters like norepinephrine, serotonin, and those involved in central sensitization. nVNS was reported to have a reduction in headache ache days along with adverse effects such as respiratory tract infections and gastrointestinal disorders. The Prospective Study of nVNS for the Acute Treatment of Migraine (PRESTO) showed pain reduction at 120 minutes (40.8% with nVNS and 27.6% with sham, P = 0.030) as well as pain freedom at 30 minutes. Being noninvasive, the device can be easily used and is safe [188].

## Conclusions

Migraine is a primary headache disorder that is prevalent across all age groups and has a significant disability burden. Being familiar with the evolving areas of migraine epidemiology, pathophysiology, diagnosis, and treatment modalities can assist physicians across the globe in narrowing down the diagnosis, planning the treatment options, reducing disabilities due to migraine, and improving the quality of life of patients. Currently, migraine can be managed with various treatment options comprising both non-pharmacological and pharmacotherapy approaches. Stratified treatment is an evolving method preferred over step treatment to abort acute attacks offering quick headache resolution. Newer drugs, FDA-approved or currently in various phases of clinical trials, acting on the CGRP pathway have great potential to be added to the routine clinical armamentarium. Neuromodulation is potentially emerging as a second-line option in patients non-responsive to standard drug therapy. Scientists have made major strides recently to narrow the diagnosis and individualize the treatment of migraine. Owing to these advancements, we can expect a paradigm shift in approach to the management of migraine and improving the quality of life of the afflicted patients.
